# Intrinsically topological second Chern insulator via synthetic dimensions

**DOI:** 10.1093/nsr/nwae137

**Published:** 2024-04-08

**Authors:** Seokwoo Kim, Junsuk Rho

**Affiliations:** Department of Mechanical Engineering, Pohang University of Science and Technology (POSTECH), South Korea; Department of Mechanical Engineering, Pohang University of Science and Technology (POSTECH), South Korea; Department of Chemical Engineering, Pohang University of Science and Technology (POSTECH), South Korea; Department of Electrical Engineering, Pohang University of Science and Technology (POSTECH), South Korea

## Abstract

Exploring Hidden Dimensions: Unveiling Topological Crystals in a 4D Space.

Topology, an invariant quantity under continuous deformation, has significantly expanded our understanding of the phases of matter over recent decades. This concept, extending from quantum mechanics to classical wave platforms, prompts us to consider the interplay between various symmetry operations and the physical boundaries of lattices. In crystal bulk bands, non-trivial topology ensures the existence of local boundary modes that are protected by global symmetry, a principle known as bulk-boundary correspondence. For instance, the Chern insulator exhibits unidirectional chiral edge states, as its bulk bands become twisted evident in a nonzero Chern number defined in 2D momentum space. Traditionally, discussions of topology centered on 2D or 3D Bloch momentum space [1,2]; however, they can be expanded to higher dimensions, incorporating additional synthetic dimensions such as external fields and geometric parameters [3].

The study of band topology beyond the dimensions of the real world has been successfully demonstrated in several research projects, e.g. the 4D generalization of the quantum Hall effect [4]. In the 4D case, the band topology is characterized by the second Chern number, which is different from the first Chern number in the conventional 2D momentum space. Although the second Chern insulator has been demonstrated in various classical wave platforms, such as acoustics and photonics, the realization of the proposed complicated systems remains a challenging task.

Recently, a remarkable study reported in the *National Science Review* introduces a novel approach for designing a second Chern crystal within a 4D synthetic parametric space [5]. They explore a four-dimensional parameter space that includes two Bloch momentum spaces (${{k}_x},{{k}_y}$) and two additional synthetic translation dimensions ($\Delta x,\Delta y$) (Fig. 1). Interestingly, their crystal's topology is ‘inherently’ non-trivial, regardless of crystalline configuration. Since the bulk bands in synthetic translation space possess a non-trivial topology, it renders the Chern crystal topologically non-trivial in any configuration. This leads to the observation of a gapless 2D surface mode and robust 1D dislocation modes in real space (Fig. 1). These topological boundary modes originate from the intrinsically non-trivial topology of the second Chern insulator.

**Figure 1.**
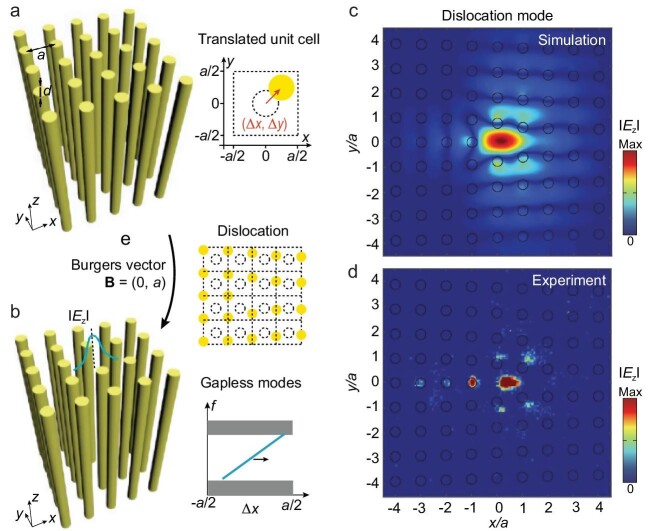
Second Chern crystal in 4D synthetic parametric space and its in-gap dislocation modes. (a) The two-dimensional periodic square lattice of dielectric rods. The unit cell is translated by (${\boldsymbol{\Delta x}},{\boldsymbol{\ \Delta y}}$) and this becomes two additional degree of freedom constituting synthetic parametric space. (b) The dislocation lattice of dielectric rods ensures the existence of gapless surface modes. (c and d) The simulated and measured in-gap 0D dislocation mode at the frequency of ${\boldsymbol{f}}$ = 9.2 GHz. Adapted from Ref. [5].

In summary, the research demonstrates topologically protected modes on lower-dimensional boundaries of this crystal, achieved through dimension reduction. This method confirms the robustness of one-dimensional gapless dislocation modes experimentally. The findings not only offer new insights into topologically non-trivial crystals with synthetic dimension but also pave the way for feasible designs in classical wave devices. It provides practical pathways for realizing high-dimensional topological states in classical wave systems, circumventing real-space dimensionality limitations. It underscores the potential of synthetic dimensions in unraveling complex topological phenomena, heralding a new era of exploration in photonic and other classical wave systems.
